# Biofortification of Wheat Cultivars to Combat Zinc Deficiency

**DOI:** 10.3389/fpls.2017.00281

**Published:** 2017-03-14

**Authors:** Muhammad U. Chattha, Muhammad U. Hassan, Imran Khan, Muhammad B. Chattha, Athar Mahmood, Muhammad U. Chattha, Muhammad Nawaz, Muhammad N. Subhani, Mina Kharal, Sadia Khan

**Affiliations:** ^1^Department of Agronomy, University of Agriculture Faisalabad, FaisalabadPakistan; ^2^Institute of Agricultural Sciences, University of the PunjabLahore, Pakistan; ^3^Department of Agronomy, Bahauddin Zakariya UniversityMultan, Pakistan; ^4^College of Agriculture, Bahadur Campus Layyah, Bahauddin Zakariya UniversityMultan, Pakistan; ^5^Department of Management Sciences, National Textile UniversityFaisalabad, Pakistan; ^6^Department of Agriculture, Government of PunjabLahore, Pakistan

**Keywords:** zinc deficiency, zinc application methods, grain zinc contents, grain phytic acid, biofortification

## Abstract

Zinc (Zn) deficiency caused by inadequate dietary intake is a global nutritional problem, particularly in developing countries. Therefore, zinc biofortification of wheat and other cereal crops is being urgently addressed and highly prioritized as a research topic. A field study was planned to evaluate the influence of zinc application on grain yield, grain zinc content, and grain phytic acid concentrations of wheat cultivars, and the relationships between these parameters. Three wheat cultivars, C_1_ = Faisalabad-2008, C_2_ = Punjab-2011, and C_3_ = Millet-2011 were tested with five different methods of zinc application: T_1_ = control, T_2_ = seed priming, T_3_ = soil application, T_4_ = foliar application, and T_5_ = soil + foliar application. It was found that grain yield and grain zinc were positively correlated, whereas, grain phytic acid and grain zinc were significantly negatively correlated. Results also revealed that T_5_, T_3_, and T_4_ considerably increased grain yield; however, T_2_ only slightly enhanced grain yield. Grain zinc concentration increased from 33.1 and 33.7 mg kg^−1^ in T_1_ to 62.3 and 63.1 mg kg^−1^ in T_5_ in 2013–2014 and 2014–2015, respectively. In particular, T_5_ markedly decreased grain phytic acid content; however, maximum concentration was recorded in T_1_. Moreover, all the tested cultivars exhibited considerable variation in grain yield, grain zinc, and grain phytic acid content. In conclusion, T_5_ was found to be most suitable for both optimum grain yield and grain biofortification of wheat.

## Introduction

Zinc (Zn) is an essential micronutrient in biological metabolism, and is receiving growing attention around the globe because of increasing reports of zinc deficiency in food crops as well as in humans (Alloway, 2004; Hotz and Brown, 2004; Cakmak, 2008). Zinc is required for normal growth and development of humans and plants (Hafeez et al., 2013). Moreover, it affects multiple aspects of the immune system (Shankar and Prasad, 1998) and is required for normal development and proper function of cell mediating immunity, neutrophils, and natural killer cells (Prasad, 2008). Similarly, in plants, zinc plays a crucial role in enzymatically driven metabolism (Tisdale et al., 1984). It also makes a notable contribution toward gene expression, stress tolerance (Cakmak, 2000), and pollen tube formation (Pandey et al., 2006).

Zinc deficiency is among the top five micronutrient deficiencies and severely affects one-third of the world’s population, especially rural communities (Hotz and Brown, 2004; Stein, 2010). Inadequate intake of food low in zinc content is a major contributor to the prevalence of zinc deficiency in humans. As one of the commonest cereal crops, wheat contributes to the provision of daily calories, proteins, and bioavailable micronutrients. In many developing nations, wheat provides over 50% of the daily calorific intake (Cakmak, 2008).

An excessive intake of monotonous wheat products is a major reason for zinc malnutrition in humans because wheat is inherently low in zinc content and high in phytate, which further limits zinc bioavailability (Welch and Graham, 2004; Cakmak et al., 2010b). Different reports are available indicating that more than 50% of wheat around the globe is cultivated on zinc-deficient soils (Alloway, 2004; Cakmak, 2008), which further lowers grain zinc content. The adoption of high-yielding cultivars seems to have aggravated this problem (Zhao and McGrath, 2009; Cakmak et al., 2010b; Stein, 2010). Furthermore, wheat processing after harvesting markedly decreases grain zinc and micronutrients such as iron, which enhances the chance of zinc deficiency in humans (Cakmak, 2008; Zhang et al., 2010b; Kutman et al., 2011). Hence, there is an urgent challenge and dire need to increase grain zinc content and bioavailability in developing countries (Welch and Graham, 2004; Cakmak, 2008; Zhao and McGrath, 2009).

In response to the aforementioned problem, different approaches have been suggested and applied in developing nations (Bouis, 2003; Pfeiffer and McClafferty, 2007), where the biofortification of cereals with important micronutrients is receiving a great deal of attention (Cakmak, 2008; Zhao and McGrath, 2009; Bouis and Welch, 2010). Key tools in biofortification include breeding and agronomic techniques such as fertilizer application. Breeding techniques are prime, and there are long-term strategies to deal with micronutrient malnutrition through evolving new genotypes with higher grain nutrient content (Welch and Graham, 2004; Bouis et al., 2011). However, breeding techniques take time and are costly, so agronomic techniques may provide a quicker solution to the micronutrient malnutrition problem. Agronomic techniques involve fertilizer application by seed priming or soil and foliar application. Moreover, the fertilization approach is a quick and complementary strategy, which maintains and builds a pool of zinc for translocation and uptake (Cakmak, 2008). Zinc has moderate phloem mobility (Haslett et al., 2001), so its application as a foliar feed alone or as a combination of soil plus foliar application markedly increases grain zinc content (Cakmak, 2008). Furthermore, grain zinc concentration is severely affected by the availability of a physiological pool of zinc in vegetative tissues as a result of foliar application (Cakmak et al., 2010a); the latter can substantially increase zinc concentration in wheat endosperm (Cakmak et al., 2010a; Zhang et al., 2010a). On the other hand, soil application of zinc is less effective in increasing grain zinc concentration because of poor zinc mobility and its rapid absorption in alkaline calcareous soils (Alloway, 2008). Furthermore, zinc application substantially reduces grain phytic acid concentration, which is widely used as an indicator of zinc bioavailability in diets (Erdal et al., 2002; Cakmak et al., 2010b). Therefore, agronomic biofortification through fertilization is the most valuable approach for combatting zinc malnutrition.

Zinc is an active nutrient and has antagonisms [phosphorus (Mousavi, 2011), copper (Imtiaz et al., 2003), and cadmium (Moustakas et al., 2011) and synergisms [iron (Mousavi, 2011) and boron (Rengel et al., 1998)]. Higher phosphorus levels in soil reduce zinc concentrations in plant aerial parts and also reduce total zinc content; similarly, phosphorus exerts P-Zn antagonism in plants (Singh et al., 1986). Phytic acid binds nutritionally important minerals such as zinc and impairs their biological utilization. Thus, a high concentration of phytic acid in cereal-based foods is a major cause of zinc deficiency in humans (Gibson et al., 1997). To combat this, the application of zinc substantially reduces grain phytic acid content and increases zinc bioavailability, as shown in soybean after enhanced zinc supply (Raboy and Dickinson, 1984). In most cases, there is an inverse relationship between grain yield and grain zinc concentration (Garvin et al., 2006; McDonald et al., 2008) with higher grain zinc concentrations being most commonly associated with lower yielding genotypes (Oury et al., 2006; Fan et al., 2008; McDonald et al., 2008). Moreover, some studies reveal that grain yield increases simultaneously, along with a remarkable increase in grain zinc concentration, as shown in Pakistan (Zou et al., 2012), China (Karim et al., 2012), and Turkey (Yilmaz et al., 1997). Thus, this study aimed to address the following questions: (1) what is the influence of zinc application method on grain yield, grain zinc concentration, and grain phytic acid concentration of wheat, (2) what is the relationship between grain zinc concentration and grain yield, and (3) what is the relationship between grain zinc and grain phytic acid content?

## Materials and Methods

### Experimental Site and Planting Material

The experiment was conducted at the Agronomic Research Farm, University of Agriculture, Faisalabad, Pakistan, during the winter seasons (November to April) of 2013–2014 and 2014–2015. The temperature of this region ranges between −1°C in January and 48°C in June, with a mean annual rainfall of around 200–250 mm. The prevailing conditions during both years are presented in **Tables 1A,B**. Seeds from three wheat cultivars, Faisalabad-2008, Punjab-2011, and Millet-2011 were obtained from the Wheat Research Institute, Ayub Agricultural Research Institute, Faisalabad, Pakistan.

**Table 1A:** Prevailing climatic conditions for the experimental site during crop growing seasons for the years 2013–2014.

Months	Rainfall (mm)	Monthly mean maximum temperature (°C)	Monthly mean minimum temperature (°C)	Monthly average temperature (°C)	Relative humidity (%)
November-13	0.5	26.1	11.8	19	59.4
December-13	0	20.5	8.4	8.2	66.5
January-14	0	19.1	6.1	12.6	63.8
Feburary-14	14.3	20	8.9	14.4	65
March-14	41.7	24.7	13.6	19.2	60.1
April-14	28.2	32.2	18.6	25.4	52.2

**Table 1B:** Prevailing climatic conditions for the experimental site during crop growing seasons for the years 2014–2015.

Months	Rainfall (mm)	Monthly mean maximum temperature (°C)	Monthly mean minimum temperature (°C)	Monthly average temperature (°C)	Relative humidity (%)
November-14	10	26.3	11.5	18.9	61.7
December-14	0	18.5	5.9	12.2	75
January-15	12.2	16.6	6.9	11.7	75.3
Feburary-15	20.5	22	11.1	16.5	66
March-15	67.9	24.5	13.6	19.1	64
April-15	32.8	33.2	20.7	27	43.9

### Treatments and Crop Husbandry

The experiment included three different wheat cultivars, C_1_ = Faisalabad-2008, C_2_ = Punjab-2011, and C_3_ = Millet-2011, and five zinc application protocols: T_1_ = control, T_2_ = seed priming, T_3_ = soil application, T_4_ = foliar application, and T_5_ = soil + foliar application. The source of zinc was “Naya Zinc,” which is 98% pure containing 21% zinc as ZnSO_4_⋅7H_2_O. For T_1_, no zinc was applied, while in T_2_, seeds were soaked in 0.3% ZnSO_4_ solution; for T_3_, ZnSO_4_⋅7H_2_O was applied at the rate of 50 kg ZnSO_4_ per ha; for T_4_, ZnSO_4_⋅7H_2_O was applied at the rate of 0.5% at two growth stages (booting and milking); and in T_5_, zinc was applied in both the soil and as a foliar feed. Furthermore, for T_2_, seeds were initially soaked in 0.3% ZnSO_4_ solution and subsequently given three surface washings with distilled water, then dried close to the original moisture level with forced air, after which they were sealed in polythene bags and stored in a refrigerator at 7 ± 1°C until use. In T_3_, ZnSO_4_⋅7H_2_O was applied to the soil surface and after that incorporated into soil prior to sowing. For T_4_, each application of an aqueous solution of ZnSO_4_⋅7H_2_O was sprayed in the late afternoon until most leaves were wet.

The seeds were sown on November, 19 in 2013–2014 and November, 23 in 2014–2015. In both growing seasons, wheat cultivars were planted in rows 22.5 cm apart using a hand drill and a seed rate of 125 kg ha^−1^. Nitrogen, phosphorus, and potassium were applied at a rate of 100:50:50 (N:P:K) kg ha^−1^. Nitrogen, phosphorus, and potash were applied in the form of urea (46% N), single super phosphate (14% P), and sulfate of potash (50% K), respectively. Nitrogen was applied in three splits, one-third as a basal dose and the remaining two-thirds in two equal splits at the tillering and booting stages. All the potash and phosphorus were applied as basal doses. During crop growth, field water conditions were managed by flood irrigation.

### Soil Analysis

To determine the physicochemical properties of experimental soil, composite soil samples were taken from the top (0–30 cm) soil layer of the experimental site prior to sowing. Collected samples were analyzed using the protocols described by Homer and Pratt (1961). The soil was loamy containing sand (41.23%), silt (39.35%), and clay (19.42%) particles, having a bulk density of 1.36 g cm^-3^, pH 7.8, EC 1.03 dSm^−1^, organic matter 0.81%, available nitrogen 0.031%, available phosphorus 22 ppm, available potassium 121 ppm, and available zinc 29 ppm.

### Sampling and Measurements

At maturity, the crop was harvested and tied into bundles for determination of yield. The individual plots were threshed using a mini thresher. Grain weight for each treatment was recorded by digital balance in kilograms and later expressed in tons per hectare (t ha^−1^). The harvested grain was stored for determination of grain zinc and phytic acid concentration.

### Sample Preparation and Analysis

Samples of wheat grain were dried in a drying oven at 60°C for 48 h (Liu et al., 2006). Dried samples were ground in a mill (IKA Werke, MF 10 Basic, Staufen, Germany) fitted with a stainless steel chamber and blades. Subsequently, finely ground 1.0 g samples of wheat flour were placed in a conical flask and kept overnight after adding a di-acid (HNO_3_:HClO_4_ ratio of 2:1) digestion mixture (Jones and Case, 1990). After 24 h, samples were digested on a hot plate at 150°C until all the material was digested. After digestion, the material was cooled and diluted to 50 ml by adding de-ionized water. Digesta was then filtered with Whatman filter paper No. 42 and stored in air tight plastic bottles. Zinc concentration in the digested samples was determined by atomic absorption spectrophotometer (PerkinElmer, 100 AAnalyst, Waltham, MA, USA). Phytic acid in the extract was measured by an indirect method that uses absorption of the pink color developed by un-reacted Fe (III) with 2,2′-bi-pyridine (Haug and Lantzsch, 1983) at 519 nm with a spectrophotometer (Shimadzu, UV-1201, Kyoto, Japan). All samples for zinc and phytic acid determinations were prepared and analyzed in duplicate.

### Experimental Design and Statistical Analysis

The experiment was laid out in a randomized complete block design in a factorial arrangement with three replications. Data were statistically analyzed using Statistix 8.1 (Analytical, Tallahassee, FL, USA), while the least significant difference (LSD) test at 5% probability was used to compare treatment means. Graphs for experimental and climatic data were prepared using Microsoft Excel 2007.

## Results

Zinc application methods significantly (*p* ≤ 0.05) affected economic yield, grain zinc, and grain phytic acid concentrations (see **Table 2**). Maximum improvement in grain yield, 24.27 and 24.06%, was recorded with T_5_ in 2013–2014 and 2014–2015, respectively, and the minimum improvement in grain yield was recorded under T_1_. The overall trend of zinc application methods regarding grain yield was: T_5_ > T_3_ > T_4_ > T_2_ > T_1_. Similarly, zinc application via different methods markedly (*p* ≤ 0.05) influenced grain zinc and phytic acid concentrations. As for grain zinc concentration, a maximum increase of 50.08 and 46.59% was observed in T_5_, followed by 47.81 and 46.59% increase in T_4_ during both years. T_5_ appeared to be an excellent strategy to increase grain zinc concentration, whereas minimum increase was observed with T_2_ and T_1_ (**Table 2**). Grain phytic acid concentration was also significantly (*p* ≤ 0.05) reduced by zinc application (**Table 2**). During 2013–2014 and 2014–2015, a reduction of 29.05 and 28.69% in grain phytic acid was recorded under T_5_ followed by T_4_ and T_3_ (**Table 2**); minimum reduction in grain phytic acid content was recorded with T_2_ and T_1_.

**Table 2:** The effect of zinc application methods on grain yield, grain zinc, and grain phytic acid concentrations of wheat cultivars.

Zinc application method	Grain yield (t ha^−1^)		Grain zinc concentration (mg kg^−1^)		Grain phytic acid concentration (mg g^−1^)	
	2013–2014	2014–2015	2013–2014	2014–2015	2013–2014	2014–2015
No zinc	3.59 e	3.66 e	33.1 e	33.7 e	11.68a	11.53a
Seed priming	3.90 d	3.99 d	38.4 d	40.5 d	11.37a	11.18a
Soil	4.61 b	4.69 b	44.4 c	47.6 c	9.68b	9.56b
Foliar	4.37 c	4.13 c	59.6 b	60.7 b	8.74c	8.66c
Soil + foliar	5.10 a	5.18 a	62.3 a	63.1 a	8.28d	8.19c
LSD (*p* ≤ 0.05)	**0.024**	**0.050**	**2.06**	**2.27**	**0.43**	**0.51**
Cultivars						
Faisalabad-2008	3.77 c	3.88 c	41.8 c	43.1 c	10.90a	10.88a
Punjab-2011	4.80 a	4.89 a	54.4 a	55.6 a	9.73b	9.53b
Millat-2011	4.34 b	4.40 b	46.5 b	48.6 b	9.21c	9.11c
LSD (*p* ≤ 0.05)	**0.031**	**0.039**	**1.59**	**1.78**	**0.33**	**0.39**

*LSD values were shown as bold in order to differentiate from data values.*

Similarly, all wheat cultivars differed significantly for grain yield, grain zinc, and phytic acid concentrations (**Table 2**). Wheat cultivar Punjab-2011 had a higher grain yield and grain zinc concentration followed by Millet-2011 and Faisalabad-2008 for both study years. Minimum grain yield and grain zinc concentrations were recorded in Faisalabad-2008 (**Table 2**). However, for grain phytic acid, considerable variation was observed among the wheat cultivars. Punjab-2011 had the lowest grain phytic acid content, followed by Millet-2011. However, Faisalabad-2008 performed poorly and had a higher grain phytic acid content when compared to Punjab-2011 and Millet-2011 (**Table 2**).

**FIGURE 1:**
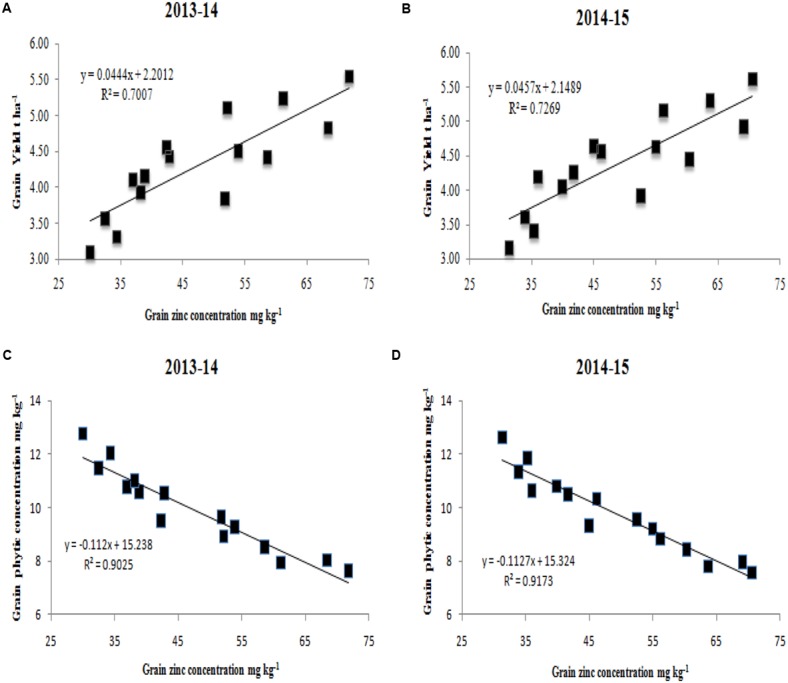
**Relationships between grain zinc concentration and grain yield (A,B)**, and grain zinc and phytic acid concentration **(C,D)** during the years 2013–2014 and 2014–2015. mg kg^−1^, milligram per kilogram; t ha^−1^, tons per hectare.

Interactions between zinc application methods and wheat cultivars were found to be significant for grain zinc concentration but not for grain yield or grain phytic acid concentration (see **Table 3**). For the interactive effect of grain zinc concentration and wheat cultivars, Punjab-2011 registered the highest values for grain zinc concentration at T_5_ in the first (71.8 mg kg^−1^) and second (70.6 mg kg^−1^) year, respectively. However, Faisalabad-2008 registered the lowest value of grain zinc concentration under T_1_ (**Table 3**). There was a significant positive correlation between grain yield and grain zinc during both years of study (**Figures 1A,B**); an increase in grain zinc concentration substantially enhanced grain yield. Similarly, and interestingly, a strong negative correlation was found between grain zinc and grain phytic acid concentration (**Figures 1C,D**); it was found that zinc enriched seeds had a lower phytic acid content than seeds with lower zinc content.

**Table 3:** Interactive effect of zinc application methods and wheat cultivar on grain yield, grain zinc, and phytic acid concentrations.

Zinc application method	Cultivars	Grain yield (t ha^−1^)		Grain zinc concentration (mg kg^−1^)		Grain phytic acid concentration (mg kg^−1^)	
		2013–2014	2014–2015	2013–2014	2014–2015	2013–2014	2014–2015
No zinc	Faisalabad-2008	3.10	3.17	30.0i	31.3i	12.8	12.6
	Punjab-2011	4.11	4.19	36.9 fg	36.0gh	10.8	10.6
	Millat-2011	3.56	3.61	32.5hi	33.9 hi	11.5	11.3
Seed priming	Faisalabad-2008	3.32	3.41	34.3gh	35.3h	12.2	12.0
	Punjab-2011	4.43	4.56	42.8d	46.2 d	10.7	10.5
	Millat-2011	3.94	4.01	38.2f	39.9fg	11.2	11.0
Soil	Faisalabad-2008	4.16	4.26	38.8 ef	41.7 ef	10.6	10.5
	Punjab-2011	5.11	5.16	52.2 c	56.2c	8.9	8.8
	Millat-2011	4.56	4.63	42.3 de	45.0 de	9.5	9.3
Foliar	Faisalabad-2008	3.85	3.92	51.8 c	52.5 c	9.7	9.6
	Punjab-2011	4.83	4.92	68.4 a	69.1 a	8.0	8.0
	Millat-2011	4.42	4.45	58.6 b	60.3 b	8.5	8.4
Soil + foliar	Faisalabad-2008	4.51	4.63	53.9 c	55.0 c	9.3	9.2
	Punjab-2011	5.55	5.62	71.8 a	70.6 a	7.6	7.6
	Millat-2011	5.24	5.3	61.2 b	63.7 b	7.9	7.8
LSD (*p* ≤ 0.05)		**NS**	**NS**	**3.57**	**3.93**	**NS**	**NS**

*LSD values were shown as bold in order to differentiate from data values.*

## Discussion

Zinc is essential for all biological systems in humans, animals, and plants. Low zinc availability and zinc fixation resulted in greater reduction of grain yield and grain zinc content; further, it also enhanced grain phytic acid content (**Table 2**).

Zinc application improves yield and yield components through various mechanisms, for example, it improves chlorophyll content and triggers photosynthetic activity and auxin synthesis which lead to better growth and development of the crop, thus effectively amplifying yield and yield components (Rakesh and Jitendra, 2014). Seed priming is a cheap source of zinc application, which can increase the yield of various crops (Harris et al., 2008); however, in the present study, T_2_ was unable to fulfill the zinc requirement of the wheat crop for optimum yield (**Table 2**). The slight improvement in grain yield with T_2_ could be explained by the fact that zinc synchronizes stand establishment and also helps in increasing the range of temperature during germination, which ultimately enhances wheat grain yield (Farooq et al., 2008). For the other application methods, T_5_ markedly enhanced grain yield as compared to T_3_ and T_4_ (**Table 2**). These results agree with previous literature (Torun et al., 2001; Zorita et al., 2001) where it is reported that foliar feeding of zinc ensures the increased availability of zinc at anthesis and grain filling stages, while Khan et al. (2009) also states that soil application substantially improves the translocation of nutrients from soil, which leads to better stand establishment and grain yield. Variation in grain yield, grain zinc, and phytic acid concentration among wheat cultivars might be due to their genetic makeup and their response toward zinc uptake.

Wheat, inherently, has a lower grain zinc concentration, especially when grown on zinc-deficient soils. Wheat cultivars are mostly zinc deficient and unable to fulfill human zinc requirements. For a measurable impact on human health, agronomic biofortification should enhance grain zinc content from 35 to 45 mg kg^−1^ (Pfeiffer and McClafferty, 2007; Cakmak, 2008). In our study T_5_, T_4_, and T_3_ significantly increased grain zinc content as compared to T_2_ and T_1_. The improvement in grain zinc concentration in T_5_ could be due to the improved availability of nutrients and maintenance of a greater zinc pool within plant tissues during the later growth stages. However, T_4_ was superior to T_3_ for improving grain zinc concentration even though just a small amount of zinc was applied in T_4_ compared to T_3_ (Erdal et al., 2002; Cakmak et al., 2010a). On the other hand, T_3_ was less effective as compared with T_5_ and T_4_ because of poor mobility and rapid adsorption of zinc in soil (Alloway, 2008). This explains why better results were obtained regarding grain zinc concentration from T_5_ (**Table 2**). Soil application was less effective for several reasons. Mostly, wheat roots and applied zinc have different soil distribution profiles, which reduces the uptake of zinc by plant roots (Holloway et al., 2010). In addition, top soil is mostly dry during the reproductive stages, meanwhile root activity is generally reduced due to lower allocation of photo-assimilates. Thus, zinc uptake from soil or zinc fertilizers is usually reduced during the reproductive stages, a factor that substantially decreases zinc accumulation in grains. Zinc accumulation in wheat grain largely depends on re-translocation of zinc from vegetative tissue during the reproductive stages (Cakmak, 2008; Cakmak et al., 2010a). Foliar feeding of zinc maintains a high concentration of zinc in vegetative tissues during re-translocation periods and contributes significantly to zinc biofortification of wheat grain under field conditions.

Phytate is a major phosphorus storing compound in cereal grains and acts as a metal chelator in the human intestine; it therefore hinders the absorption of dietary zinc and other metals into the blood (Bohn et al., 2008). According to Rengel and Graham (1995b), soil zinc deficiency enhances plant phosphorus uptake and reduces zinc availability. Zinc application decreased grain phytic acid concentrations (**Table 2**), and this may be attributed to the inhibitory effect of zinc on root uptake and the accumulation of phosphorus in plant shoots (Erdal et al., 2002). In the present study, T_5_ substantially reduced grain phytic acid concentration followed by T_4_ (**Table 2**). These results agree with previous findings of Mabesa et al. (2013). On the other hand, foliar application of zinc is useful for increasing grain zinc concentration and decreasing phytic acid concentration, which ultimately increase zinc bioavailability in both whole wheat grain and in wheat flour (Cakmak et al., 2010a; Kutman et al., 2011).

In most previous cases, authors report an inverse relationship between grain yield and grain zinc concentration (Garvin et al., 2006; McDonald et al., 2008). However, our results indicated that grain yield and grain zinc were positively correlated, resulting in a substantial yield increase (**Figures 1A,B**). These results are not consistent with previous studies of Oury et al. (2006) and McDonald et al. (2008) who reported an inverse relation between grain yield and grain zinc concentration. However, our findings support the results of Zou et al. (2012) in Pakistan, Karim et al. (2012) in China, and Yilmaz et al. (1997) in Turkey, who reported a simultaneous increase in grain yield and grain zinc concentrations with applied zinc. Considering the ever-growing global demand for food and widespread occurrence of zinc malnutrition, increasing grain Zn concentration in high-yielding wheat cultivars is important (Graham et al., 2007). In the current study, a negative correlation was also found between grain zinc and grain phytic acid content (**Figures 1C,D**). The decreasing effect of applied zinc on phytic acid content could be explained by the fact that zinc inhibits root uptake and shoot accumulation of phosphorus. It is well-reported that zinc deficiency increases the potential of plants for phosphorus uptake; however, zinc supply to zinc-deficient plants decreases phosphorus uptake and accumulation (Rengel and Graham, 1995a). Therefore, the substantial reduction in grain phytic acid content seen in **Table 2** can be attributed to zinc application reducing the uptake and accumulation of phosphorus.

## Conclusion

Zinc application via different methods substantially improved grain yield; however, seed priming had a marginal influence on grain yield. A combined application of soil + foliar zinc gave a higher grain yield on zinc-deficient soil. Similarly, maximum grain zinc concentration and lowest values for grain phytic acid were recorded in the same treatment. Therefore, the soil + foliar application of zinc was a more successful agronomic practice for achieving optimum yields, as well as grain biofortification. This study has also reported that grain yield and grain zinc were positively correlated, while grain zinc and grain phytic acid content were significantly negatively correlated.

## Author Contributions

MC (1st author) and IK designed the experiment and wrote the manuscript. MH performed the experiment. MBC and AM analyzed the data. MC (6th author), MN, and MS contributed reagents/materials/analysis tools. MK and SK revised statistical analysis and manuscript. All the authors read and approved the manuscript.

## Conflict of Interest Statement

The authors declare that the research was conducted in the absence of any commercial or financial relationships that could be construed as a potential conflict of interest. The reviewer HA declared a shared affiliation, though no other collaboration, with one of the authors MUC to the handling Editor, who ensured that the process nevertheless met the standards of a fair and objective review.
